# Protective roles of *Pyracantha fortuneana* extract on acute renal toxicity induced by cadmium chloride in rats^1^

**DOI:** 10.1590/s0102-865020190070000006

**Published:** 2019-09-12

**Authors:** Yixin Ke, Kaihang Yu, Weiliang Zeng, Guojun Lian

**Affiliations:** I Graduate student, Department of Health Inspection and Quarantine, School of Laboratory Medicine and Life Science, Wenzhou Medical University, Wenzhou, China. Conception and design of the study, acquisition of data, technical procedures, manuscript preparation and writing; II Graduate student, Department of Health Inspection and Quarantine, School of Laboratory Medicine and Life Science, Wenzhou Medical University, Wenzhou, China. Technical procedures, acquisition of data; III Graduate student, Department of Health Inspection and Quarantine, School of Laboratory Medicine and Life Science, Wenzhou Medical University, Wenzhou, China. Statistical analysis, interpretation of data; IV Associate Professor, Department of Health Inspection and Quarantine, School of Laboratory Medicine and Life Science, Wenzhou Medical University, Wenzhou, China. Conception and design of the study, acquisition of data, technical procedures, manuscript preparation and writing, final approval

**Keywords:** Pyracantha, Acute Toxicity, Cadmium Chloride, Apoptosis, Kidney, Rats

## Abstract

**Purpose:**

To investigate the protective roles of pyracantha fortune fruit extract (PFE) on acute renal toxicity induced by cadmium chloride (CdCl_2_) in rats.

**Methods:**

Rats were pretreated with PFE and consecutively injected with CdCl_2_ (6.5 mg/kg) for 5 days.

**Results:**

The concentration of Cd, kidney weight, malondialdehyde (MDA), and nitric oxide (NO) production were remarkably increased in CdCl_2_ group as well as the levels of plasma uric acid, urea, and creatinine (*P* < 0.001). However, the body weight and glutathione (GSH), superoxide dismutase (SOD), catalase (CAT), glutathione peroxidase (GPx) and glutathione peroxidase (GR) levels were markedly reduced by CdCl_2_ treatment (*P* < 0.001). Histological manifestations of renal tissue showed severely adverse changes. Moreover, CdCl_2_ treatment significantly decreased the B-cell lymphoma-2 (Bcl-2) expression while increased the Bcl-2-Associated X Protein (Bax), tumor necrosis factor-α (TNF-α) expression (*P* < 0.001). Additionally, the expression of Nrf2/Keap 1 related proteins Keap-1 gained a significant increase (*P* < 0.001), whereas the Nrf2, HO-1, γ-GCS, GSH-Px and NQO1 expression decreased by CdCl_2_ treatment (*P* < 0.05). These rats were pretreated with PFE to improve the changes caused by CdCl_2_ treatment.

**Conclusion:**

PFE could protect the kidney against acute renal toxicity induced by CdCl_2_.

## Introduction

Cadmium (Cd) is designated as an endocrine disruptor/endocrine disrupting chemical (EDC), a potential toxicant metal derived from horticultural and industrial sources^1^. It is a pollutant for most human foods because it has a high soil-plant transmission rate, making diet a main source of contact^2^. The level of Cd toxic in kidney, lung and testis tissues has been determined^3^. A previous study reported that many transporters play important roles in the accumulation of Cd in kidney tissue, including metallothionein, Cd binding proteins containing thiol (-SH) groups and divalent metal ion transporter 1^4^. Cd accumulates in kidney to produce reactive oxygen species (ROS), which leads to inflammation, oxidative stress, glomerular dysfunction and programmed cell death^5^, and interferes with basic elements such as calcium and zinc^6^. The S1 and S2 segments of the proximal convoluted tubule are the target sites of renal tissue toxicant, leading to renal dysfunction^7^. In addition, Cd is considered as the cause of lipid peroxidation and is generally considered to be the major cause of the deleterious effects on membrane-dependent function^8^, which makes the kidney more vulnerable to cadmium. Due to the lack of adequate methods for treating cadmium nephrotoxicity, there is an increasing interest in the use of antioxidants to prevent the kidney from the toxicity of Cd.

Pyracantha fortuneana *(Pfortuneana),* one of Maloideae subfamily, mainly distributed in the southwest of China^9^ rich in polyphenols such as rutin, and its hexose compound, and polymeric (epicate)-catechin (proanthocyanidin 2, PB2)^10^. In previous studies, the optimal P *fortuneana* fruit extract (PFE) is obtained via chemical antioxidant activity- guided extraction^11^ ^–^ ^13^). Previous reports showed that *P fortuneana* extracts inhibit the inflammatory reactions in mice, reduce oxidative stress, and reduce liver damage in mice by carbon tetrachloride (CCl4)^12^ ^,^ ^14^. However, it remains unclear whether PFE plays a role in cadmium-induced nephrotoxicity.

Keap1/Nrf2 is an important regulatory pathway in the process of oxidative stress. Keap1 is a blocker protein in the Kelch family, which usually presents on the cytoplasmic actin cytoskeleton and is a negative regulatory protein of Nrf2^15^. Nrf2 belongs to the Cap-n-Collar (CNC) regulatory protein family and is a key transcription factor in cellular antioxidant stress. It is inactive with Keap1 in the cytoplasm under physiological conditions. When the body is stimulated by other nucleophiles or in an oxidative stress state, Nrf2 is dissociated from Keap1, and after Nrf2 phosphorylation, it is transferred into the nucleus and binds to the antioxidant response element (ARE), which initiates ARE-regulated downstream phase II metabolic enzymes and antibiotics. The expression of the oxidized protein gene enhances the body's ability to resist oxidative stress^16^. This study aims to investigate the potential protective effects of PFE administration on acute nephrotoxicity in a rat model of cadmium exposure. The antioxidant and anti-apoptosis activities of PFE make it to achieve these antioxidant protection effects via Nrf2/Keap 1 pathway. Therefore, the use of PFE may be beneficial for renal toxicity caused by CdCl_2_.

## Methods

### Chemicals

CdCl_2_ and other chemicals for histological, biochemical analysis were obtained from Sigma (St Louis, MO, USA).

### Pyracantha fortune fruit

The pyracantha fortune fruit was collected from the Mount of Wuling (Hubei Province, China) in August 2018. A specimen is deposited in the Institute of Traditional Chinese Medicine and Natural Products, Guangzhou University of Traditional Chinese Medicine (Guangzhou, China). The pyracantha fortune fruit (air-dried, 10.0 kg) was refluxed twice with 25 L 60% (v/v, aqueous/ethanol) for 2 hours repeatedly. Then, leachate got dried under vacuum conditions and the remnants got dissolved in distilled water and stored in a closed bottle under the temperature of −20°C. The extracts were specified as pyracantha fortuneana extract (PFE)^17^.

### Animals

Thirty-two-adult male Wistar rats (7-8-week-old, weighing 150-170 g) were purchased from Guangdong Medical Lab Animal Center (Foshan, China). The rats were placed at the Animal Experimental Center of Guangzhou University of Traditional Chinese Medicine at room temperature with 12-h light/dark cycles. Rats were able to obtain granular rodent feed and water and were randomly divided into control group [intraperitoneally (i.p.) injected, 0.9% NaCl (physiological saline) daily for 5 days], CdCl_2_ group (injected i.p., 6.5 mg/kg CdCl_2_ daily for 5 days), PFE group (orally administered, 250 mg/ kg) and PFE + CdCl_2_ group (pretreatment administered 250 mg/kg PFE 1 h before injecting 6.5 mg/kg CdCl_2_ i.p. daily for 5 days) (n = 8 in each group)^18^. Rats were euthanized after a final dose for 24 hours (beheading). A 10% (w/v) homogenate was prepared for analysis. Briefly the kidneys of rats were dissected, weighed, and immediately homogenized in 10 mM phosphate buffer (pH 7.4) with ice-cold. All animal experiments were accordant with the Committee on Research Ethics for Laboratory Animal Care at the Animal Experimental Center of Guangzhou University of Traditional Chinese Medicine, and approved by the National Institutes of Health (NIH) Guidelines for the Care and Use of Laboratory Animals (8th edition).

### Cd concentration in renal tissue

Kidney tissue specimens were simultaneously subjected to heavy and wet crushing for 2 hours with 1 M nitric acid in the same time. The ashed specimens were diluted to 50 ml with deionized water. The metal content was measured in 22.8 nm graphite furnaces using atomic absorption spectrophotometry (Perkin-Elmer 3100, Thermo Fisher Scientific, USA). The values of Cd were expressed as micrograms per gram of wet kidney tissue.

### Histology

The specimens of kidney tissue were selected and fixed with 10% formaldehyde in PBS at room temperature for 24 h. The specimens were embedded in paraffin, sliced at 4-5 μm thickness, and then dyed with haematoxylin and eosin (H&E). The histological samples were evaluated by two investigators who were blinded to the applied power settings. Then, using light microscopy for analysis the histological characteristics and inflammatory status of glomerular and tubules.

### ELISA assay

Elisa was performed using homogenates from tissue samples, which were collected from the cranial pole (whatever the part of the kidney you used) of the kidney, weighed, and immediately homogenized in 10 mM phosphate buffer (pH 7.4) with ice-cold. Malondialdehyde (MDA), nitric oxide (NO), glutathione (GSH), catalase (CAT), superoxide dismutase (SOD), glutathione peroxidase (GPx) and glutathione reductase (GR) activities were measured according to manufacturer's Instructions by ELISA kits (Thermo Fisher Scientific, USA). A spectrophotometer was used to read the absorbance of each well at 450 nm, and the contents of each well were calculated by using a standard curve.

### qRT-PCR

Kidney tissue was dissected soon enough on ice and the total RNA was extracted by the Trizol reagent (Invitrogen, Carlsbad, CA). Then, the SuperScript RT kit from Invitrogen (Invitrogen, Carlsbad, CA) was used to reverse transcribed 2μg of total RNA from samples to cDNA. ABI PRISM7900 Sequence Detection System (Applied Biosystems, USA) with PowerUp™ SYBR® Green Master Mix (Thermo Fisher Scientific, USA) was used to perform Quantitative RT-PCR. Primer sequences were listed in Table 1. 2^-ΔΔCt^ method was used for analysis of the relative expression levels of mRNA, and GAPDH served as internal control.

**Table 1:** The sequences of mRNA primers.

ID	Forward (5’ ~ 3’)	Reverse (5’ ~ 3’)
Bcl-2	CTGGTGGACAACATCGCTCTG	GGTCTGCTGACCTCACTTGTG
Bax	GGCGAATTGGCGATGAACTG	GGCGAATTGGCGATGAACTG
TNF-α	CCTCTTCTCATTCCTGCTC	CTTCTCCTCCTTGTTGGG
GAPDH	AAGGAAATGAATGGGCAGCC	TAGGAAAAGCATCACCCGGA

### Western blotting

According to the manufacturer's instructions, the total protein in the cells or tissue was extracted using the RIPA buffer. 30ug of total proteins from each sample were separated by sodium dodecyl sulfate/polyacrylamide gel electrophoresis (SDS-PAGE) and then transferred to the polyvinylidene fluoride membrane through a wet transfer system. After blocking the membranes, they were incubated with the primary antibodies against Bcl-2, Bax, TNF-α, Nrf2, Keap1, HO-1, GSH-Px, γ-GCS and NQO1 (all 1:800, Santa Cruz, USA). β-actin (1:5000, Sigma, USA) served as a loading control. Horseradish peroxidase (HRP)-labeled secondary antibody (1:1000, Sigma, USA) was used and incubated for 1 h at 25°C. The band densities were quantified by the LICOR Odyssey infrared imaging system (LICOR Bio- science, Nebraska, USA).

### Statistical analyses

GraphPad 8.0 was used to analyze data. All data were repeated as independent experiment three times and presented as mean ± standard deviation (SD). ANOVA was used to analyze the significance of differentiation among experimental groups. (*P < 0.05, **P < 0.01, ***P <0.001) Statistical significance was verified when P < 0.05.

## Results

### PFE attenuated CdCl_2_ accumulation and damage in kidney tissues of rats pretreated with CdCl_2_

**Figure 1:**
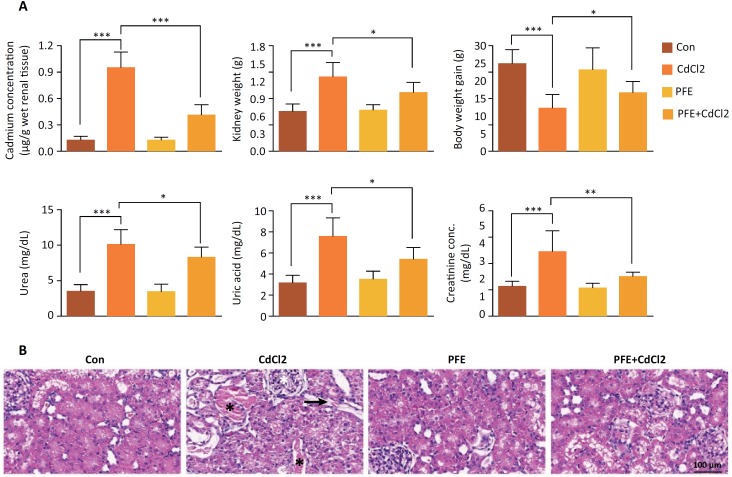
The effects of PFE on kidney tissues of rats treated with CdCl_2_. (**A**) Effect of PFE on the accumulation of Cd in renal tissue, kidney weights, body weights, uric acid, urea, and creatinine plasma levels of rats treated with CdCl_2_, ***P < 0.001 vs. Con group, *P < 0.05, ***P < 0.001 vs. CdCl_2_ group. (**B**) Histological characteristics of glomerular and tubules in control rats, CdCl_2_-treated rats, PFE-treated rats and the rats where PFE was administered before CdCl_2_, vacuoles (black arrows), inflammation and extensive degeneration *(black star)* (Scan bar, x400).

Compared to control rats, CdCl_2_ injection in rats markedly increased the Cd concentration in renal tissue *(P* < 0.001), while the increased concentration of CdCl_2_ was markedly decreased by pretreatment of PFE to rats injected with CdCl_2_ (P < 0.001) (Fig. 1A). Moreover, the kidney weight of rats exposed to CdCl_2_ alone was markedly higher than that of control rats (*P* < 0.001). However, pretreatment with PFE significantly reduced the increase in kidney weight, making it approximate to that of control group *(P* < 0.01) (Fig. 1A). However, CdCl_2_ treatment decreased the body weight gain of rats *(P* < 0.001), while the preadministration of PFE increased the value compared to that of control rats (*P* < 0.05) (Fig. 1A). Compared with the control group, the oxidative stress in rats injected with CdCl_2_ showed a higher level of serum uric acid, urea and creatinine, revealing acute renal dysfunction, compared to that of control group (Fig. 1A). Pretreatment of PFE significantly reduced these metabolite increases (*P* < 0.001). In addition, the glomerular and renal tubules in the control group showed normal microstructure characteristics, while CdCl_2_ treated rats had vacuoles (black arrows), and was accompanied by severe inflammation and extensive degeneration (black star). PFE treatment rats had the normal microstructure of renal tubules, similar to histological in the control group. The renal tubules in the rats given PFE before CdCl_2_ showed normal histological structure. However, the vacuoles in some areas were still visible (Fig. 1B). Pretreatment of PFE to CdCl_2_- injected rats significantly increased the histological characteristics in the renal tissue.

### PFE pretreatment alleviated oxidative stress markers in CdCl_2_-injected rats

**Figure 2:**
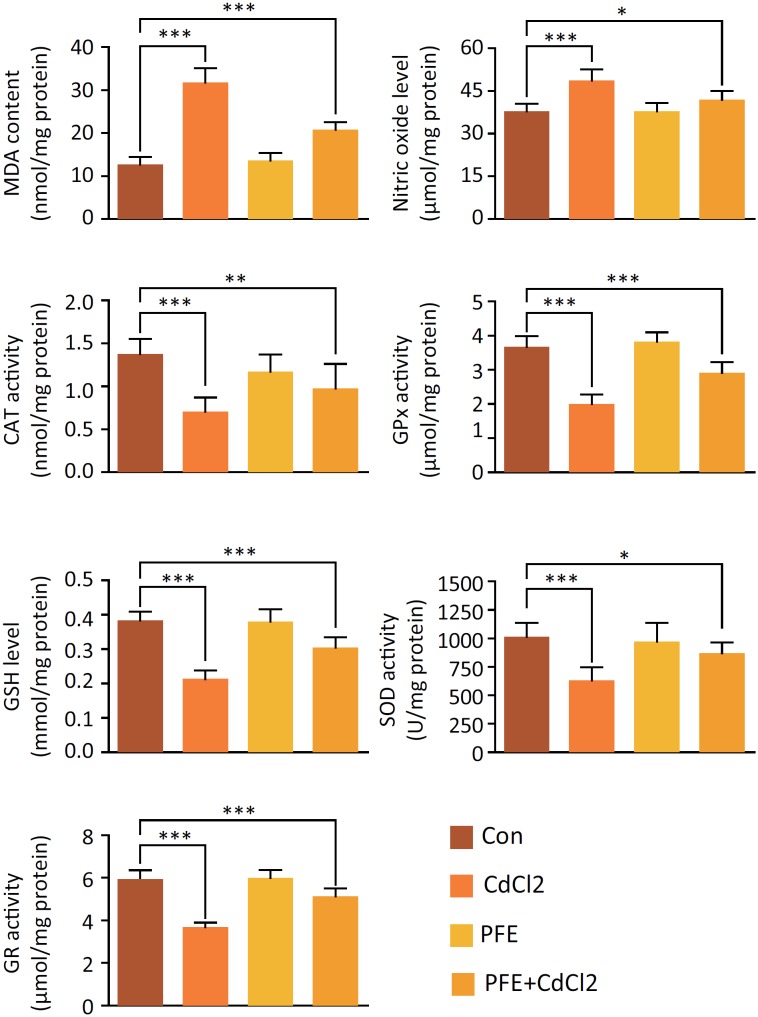
Effects of PFE pretreatment on oxidative stress markers in rats treated with CdCl_2_. Analysis of MDA content, nitric oxide level, the SOD, CAT, GPx and GR activities in the renal tissue of control rats, CdCl_2_-treated rats, PFE-treated rats and the rats where PFE was administered before CdCl_2_, ***P < 0.001 vs. Con group, **P< 0.01, ***P< 0.001 *vs.* CdCl_2_ group.

CdCl_2_ treatment in rats markedly increased the yield of nitric oxide and MDA in the renal tissue (*P* < 0.001). Rats with oxidative stress induced by CdCl_2_ showed acute renal injury, which was proved by the significant decrease of GSH content in tissues (*P* < 0.001) (Fig. 2). CdCl_2_-injection in rats also markedly decreased the antioxidant enzymes, SOD, CAT, GPx and GR activities (*P* < 0.001). Pretreatment with PFE markedly decreased the yield of MDA (*P* < 0.001) and nitric oxide (*P* < 0.05) in the renal tissue and increased the level of GSH *(P* < 0.001), which were caused by CdCl_2_ injection. Furthermore, pretreatment of PFE increased the SOD (*P* < 0.05), GPx (*P* < 0.001), CAT (*P* < 0.01), and GR (*P* < 0.001) activities compared with the enzyme activities in the CdCl_2_-treated group.

### PFE pretreatment attenuated inflammatory and apoptosis in rats treated with CdCl_2_

**Figure 3:**
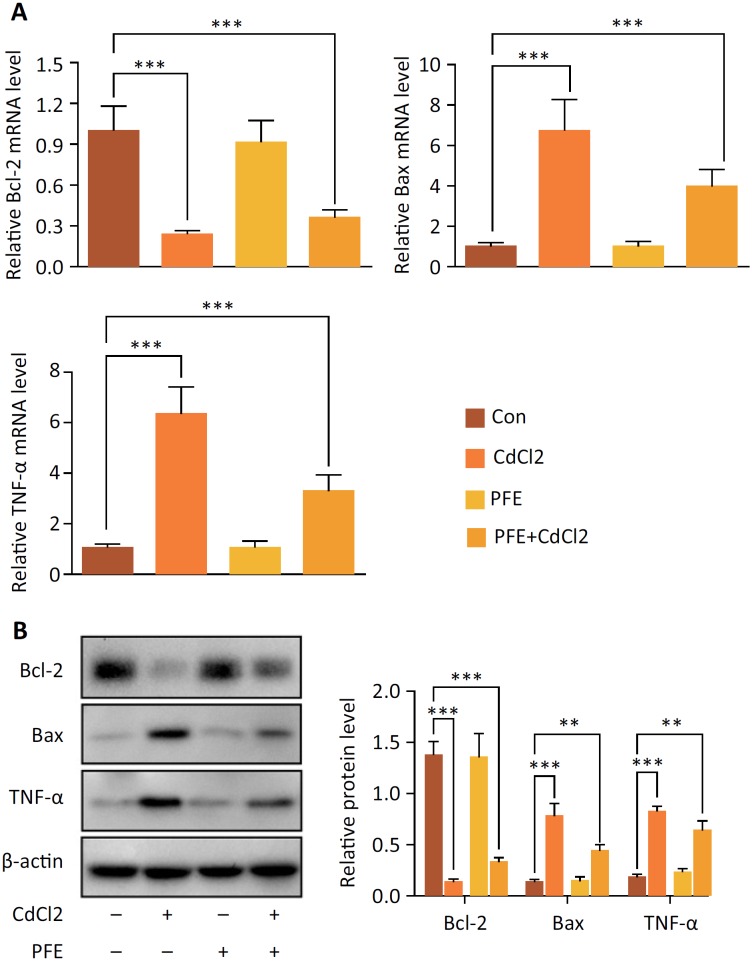
The effects of PFE on the Bcl-2, Bax, and TNF-α genes expression in the renal tissue of rats treated with CdCl_2_. (**A**) The Bcl-2, Bax, and TNF-α mRNA expression was evaluated by qRT-PCR, ***P< 0.001 *vs.* Con group, *P < 0.05, ***P < 0.001 *vs.* CdCl_2_ group. (**B**) The Bcl-2, Bax and TNF-α protein expression was evaluated by western blot, ***P < 0.001 vs. Con group, *P < 0.05, **P < 0.01 vs. CdCl_2_ group.

To detect whether the protective effects of PFE were due to its anti-inflammatory and anti-apoptotic characteristics, the Bcl-2, Bax, and TNF-α levels in the renal tissue were evaluated by qRT-PCR and western blot. Down-regulation of the Bcl-2 mRNA expression (*P* < 0.001) and up-regulation of Bax and TNF-α mRNA expression were observed in the CdCl_2_ treatment group *(P* < 0.001). However, these effects were significantly improved in the PFE pretreatment to CdCl_2_-injected rats (*P* < 0.001) (Fig. 3A). The results confirmed protective effects of PFE in the kidney of rats treated with CdCl_2_. In the rats treated with CdCl_2_, the Bax and TNF-α protein expression increased and the protein expression of Bcl-2 was reduced (*P* < 0.001). PFE pretreatment to CdCl_2_-injected rats decreased the Bax and TNF-α protein expression, while increased Bcl-2 expression in the renal tissue as compared to those expression in the CdCl_2_-injected rats (Fig. 3B).

### PFE regulated Nrf2-Keap1 signaling pathway in rats treated with CdCl_2_

**Figure 4:**
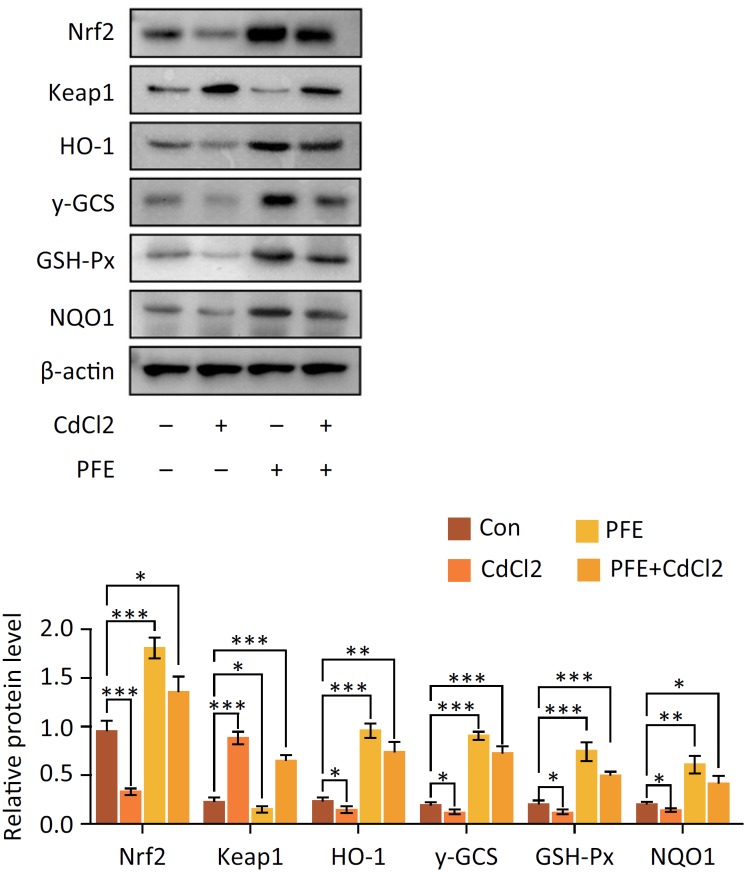
The effects of PFE on Nrf2-Keap1 signaling pathway in rats treated with CdCl_2_. The protein expression of Nrf2, Keap1, HO-1, γ-GCS, GSH-Px and NQO1 was evaluated by western blot, *P < 0.05, ***P < 0.001 *vs.* Con group, **P < 0.01, ***P < 0.001 vs. CdCl_2_ group.

The Nrf2, Keap1, HO-1, γ-GCS, GSH-Px and NQO1 protein expression in the renal tissues of all groups was evaluated by western blot (Fig. 4). Compared with the control rats, the expression level of Keap 1 protein in CdCl_2_-injected group significantly increased *(P* < 0.001), while the expression level of Nrf2, HO-1, γ-GCS, GSH-Px and NQO1 protein profoundly decreased. PFE pretreatment to CdCl_2_-injected rats markedly reduced the Keap-1 level (*P* < 0.05), while increased the Nrf2, HO-1, γ-GCS, GSH-Px and NQO1 level (*P* < 0.05) in the renal tissue as compared to the CdCl_2_-injected rats.

## Discussion

Pyracantha fortune fruit extract (PFE) is characterized as an excellent antioxidant^11^, rich in polyphenols such as rutin, hexose compound, and polymeric (epicate)-catechin (proanthocyanidin 2, PB2)^10^. A growing number of evidences support the biological properties of PFE against antioxidants, anti-tumor and protection of liver damage^11^ ^–^ ^13^. Several studies in animal models showed that Cd accumulates mainly in kidney tissue^19^ ^,^ ^20^. Some studies showed that Cd therapy leads to kidney atrophy^21^, while other studies reported kidney hypertrophy after Cd therapy^22^. However, it is still unclear whether the damage caused by heavy metals can be alleviated by *P fortuneana.* In this study, the effects of PFE on kidney injury in CdCl_2_-injected rats were investigated. The results showed that even if CdCl_2_ levels in the renal tissue were high, pretreatment with PFE in rats would significantly reduce the levels. Increasing kidney weights and the loss of body weight in CdCl_2_-injected rats observed in this investigation, was consistent with the results of previous reports^5^ ^,^ ^23^. PFE pretreatment in rats prevented these changes. We also examined markers that denote renal function and found that CdCl_2_ induced an increase in serum uric acid, urea and creatinine, and the results showed renal insufficiency. PFE pretreatment in rats decreased these indicators levels of renal dysfunction. Histological results confirmed the effect mentioned earlier, whether it was the destructive effects of CdCl_2_ and the protective effects of PFE^24^. PFE pretreatment in rats helped protect the kidneys from this damage, for example, by chelating CdCl_2_. Our results reinforced the hypothesis that PFE can attenuate CdCl_2_ accumulation and renal damage in kidney tissues of rats treated with CdCl_2_.

Malondialdehyde (MDA) is a prominent marker of oxidative stress, which reacts with proteins, DNA, and RNA in cells, leading to damage to kidney tissue^25^. Increased production of nitric oxide (NO) may activate NF-κB which increase the proinflammatory capacity of CdCl_2_, thus promoting the inducible NO synthase in macrophages^26^. Glutathione (GSH) exists both intracellularly and extracellularly in biological and allogeneic and reactive oxygen species (ROS)^27^. In recent years, several important catalytic enzymes that use GSH in defense mechanisms, such as glutathione S-transferase (GST) and glutathione reductase (GR), have shown different active patterns and injury treatments in leafy spurge tissue exposed to cold, drought and wounding treatments^28^. Superoxide dismutase (SOD) can catalyze the conversion of superoxide anions into H_2_O_2_ by a dismutation reaction^29^. Catalase (CAT) is an important antioxidant enzyme with heme as the active site prosthesis group^30^. In our current study, PFE, appears to reduce the CdCl_2_-induced renal toxicity by reducing the ability of MDA. Moreover, phenols have been reported to specifically remove NO^31^, which would clarify why PFE was so effective in reducing NO. The increase of GSH content in rat kidneys pretreated with PFE indicated that the PFE protects GSH from the decrease in the kidneys. The study found that the SOD activity of antioxidant enzymes in renal tissue of rats treated with CdCl_2_ significantly decreased. The CdCl_2_-intoxicated rats significant inhibited the free radical capture enzyme sod and cat activity. Moreover, CdCl_2_ did not activate GPX and GR. The depletion of GSH content and increased MDA levels led to a decrease in GPX and GR activity during CdCl_2_ poisoning. PFE pretreatment prevented each protein from being altered, suggesting that PFE had a protective effect against oxidase, which may be due to the scavenging activity of free radical of the PFE.

Previous studies reported that initiation of the Bax and Bcl-2 are the most noteworthy way to relate to apoptotic signals activated in vitro^32^. TNF-α is a cytokine produced by activated macrophages to respond to pathogens and other harmful stimuli and is a necessary factor for local and systemic inflammation^18^. In addition, TNF-α amplifies and prolongs the inflammatory reactions through triggering other cells to release cytokines such as interleukin −1β and media such as NO and ROS, all of which promote further inflammation and tissue damage^33^. In the current study, both the mRNA and protein level of Bax and TNF-α increased in the kidney tissue, while mRNA and protein level of Bcl-2 reduced. PFE pretreatment in rats decreased the apoptosis in the kidney. In view of the ability of various plant chemicals to protect the apoptosis of nerve cells, the protective effect of PFE on renal tissue is related to inhibiting apoptosis. Furthermore, PFE pretreatment downregulated the TNF-α mRNA and protein expression compared to that of the CdCl_2_-injected rats’ group, demonstrating the anti-inflammatory effects of PFE.

The SOD and GSH of antioxidation system can be regulated by the Nrf2/Keap1 cascade response signaling pathway^34^. The nuclear factor Nrf2 not only limits the activity of antioxidant, but also plays an important role in responding to various physiological and pathological stresses^35^ ^,^ ^36^. The degradation of NRF2 is caused by Keap1/Cul3 ubiquitin-linked enzymes^34^. In the current study, the Keap-1 protein expression level was markedly increased, while the Nrf2, HO-1, γ-GCS, GSH- Px and NQO1 protein expression level was reduced in the CdCl_2_-injected group when compared with control rats. In addition, we found that the level of Nrf2, HO-1, γ-GCS, GSH-Px and NQO1 were elevated and the level of Keap 1 was decreased by pretreatment with PFE, which was consistent with the changes of SOD activity and GSH level. The protective effect of PFE on kidney tissue is associated with inhibition of inflammation, in light of the capacity of various phytochemicals to protect against stress-induced inflammation. Interestingly, PFE enhanced the kidney by suppressing the effects of CdCl_2_. Therefore, the expression levels of Nrf2, HO-1, y-GCS, GSH-Px and NQO-1 were increased in PFE + CdCl_2_ group. These data further confirmed that the protective effect of PFE therapy on oxidative stress may be through intervention in Nrf2/Keap 1 pathway.

## Conclusions

The pretreatment of PFE suggested protective roles against CdCl_2_-induced toxicity of kidney. The antioxidant and anti-apoptosis activity of PFE can achieve these antioxidant protection effects via the Nrf2/Keap 1 pathway. Therefore, the use of PFE may be beneficial for renal toxicity caused by CdCl_2_.
